# Generation of Neurospheres from Human Adipose-Derived Stem Cells

**DOI:** 10.1155/2015/743714

**Published:** 2015-02-26

**Authors:** Erfang Yang, Na Liu, Yingxin Tang, Yang Hu, Ping Zhang, Chao Pan, Shasha Dong, Youping Zhang, Zhouping Tang

**Affiliations:** Department of Neurology, Tongji Hospital, Tongji Medical College, Huazhong University of Science and Technology, Wuhan, Hubei 430030, China

## Abstract

Transplantation of neural stem cells (NSCs) to treat neurodegenerative disease shows promise; however, the clinical application of NSCs is limited by the invasive procurement and ethical concerns. Adipose-derived stem cells (ADSCs) are a source of multipotent stem cells that can self-renew and differentiate into various kinds of cells; this study intends to generate neurospheres from human ADSCs by culturing ADSCs on uncoated culture flasks in serum-free neurobasal medium supplemented with B27, basic fibroblast growth factor (bFGF), and epidermal growth factor (EGF); the ADSCs-derived neurospheres were terminally differentiated after growth factor withdrawal. Expression of Nestin, NeuN, MAP2, and GFAP in ADSCs and terminally differentiated neurospheres was shown by quantitative reverse transcription-polymerase chain reaction (qRT-PCR), western blotting, and immunocytochemistry; cell proliferation in neurospheres was evaluated by cell cycle analyses, immunostaining, and flow cytometry. These data strongly support the conclusion that human ADSCs can successfully differentiate into neurospheres efficiently on uncoated culture flasks, which present similar molecular marker pattern and proliferative ability with NSCs derived from embryonic and adult brain tissues. Therefore, human ADSCs may be an ideal alternative source of stem cells for the treatment of neurodegenerative diseases.

## 1. Introduction

Human neurodegenerative diseases such as Alzheimer's disease [1], Huntington's disease, Parkinson's disease, amyotrophic lateral sclerosis [2], and spinal muscular atrophy [3] are characterized by a loss of neurons and glia in the brain or spinal cord. Currently, the effective way to replace neural tissue lost is through transplantation of neural stem cells (NSCs). In the past two decades, researchers have isolated NSCs [4] that have the potential to differentiate into neurons, astrocytes, and oligodendrocytes successfully from brain and spinal cord [5, 6]. Studies have indicated that the transplantation of NSCs can provide functional improvement in vivo [5, 6]. However, their procurement and low number upon harvest make them limited for clinical applications [7]. For these reasons, many researchers begin to investigate alternative types of cell that have NSCs properties.

Zuk et al. found adipose-derived stem cells (ADSCs) from human adipose tissue for the first time in 2001, which could be maintained in vitro with stable population doubling and multipotent capacity of cell differentiation [8]. A substantial amount of evidence demonstrates that, under appropriate conditions, ADSCs can selectively differentiate not only into mesenchymal lineages, but also into endodermal and ectodermal cell lineages in vitro, such as osteoblasts, chondrocytes, adipocytes, myocytes, and neural cells [9]. Human adipose tissue is ubiquitous and easily attainable in large quantities using a relatively noninvasive method. In addition, the cell number obtained from adipose tissue is usually adequate for transplantation [10]. Recent research attention has focused on the ability to selectively induce ADSCs into NSCs in vitro [11–15], which provides new scenarios for developing innovative approaches for cellular therapies of neurodegenerative diseases.

In the present study, we isolated human ADSCs from the abdominal subcutaneous adipose tissue of obese women. We described the cell population characteristics of cell surface markers and achieved the differentiation to osteogenic, adipogenic, and neurogenic lineages, demonstrating the multipotency of human ADSCs. Our study also aimed to investigate the generation of neurospheres from human ADSCs for cellular therapies of neurodegenerative diseases.

## 2. Materials and Methods

### 2.1. Isolation of Human ADSCs and Cell Culture

Human adipose tissue was obtained from the abdominal subcutaneous tissue of obese women (age range, 21 to 38 years) whose babies were delivered by cesarean section in the Maternity Department of Tongji Hospital affiliated to Tongji Medical College of Huazhong University of Science and Technology. The volunteers were all healthy and were not taking any regular medication. Before the experiments, the subjects were informed of the objectives, requirements, and procedures of the experiments. After providing informed written consent to participate in the study, a ~5 g adipose tissue was obtained from each subject. The research protocol was approved by the Ethics Committee of Tongji Hospital.

The adipose sample was washed extensively with sterile phosphate-buffered saline (PBS) (Hyclone) to remove contaminating blood cells. This step was repeated 5-6 times or until the PBS wash color was clear. Then, the washed tissue was transferred to a new sterile 60 mm^2^ culture dish (Corning), cut into 1 mm^3^ pieces, digested with 1% collagenase Type I (Invitrogen) at 37°C for 60 min, and manually shaken vigorously for 5–10 s every 15 min during the digestion. The tissue mixture was diluted with an equal volume of basal growth medium, consisting of Dulbecco's modified Eagle medium/Ham's F12 (DMEM/F12; Hyclone) supplemented with 1% penicillin/streptomycin (Solarbio), 1% l-glutamine (Genom), and 10% fetal bovine serum (FBS) (Gibco) to neutralize the collagenase activity. The mixture was centrifuged at 1200 rpm for 10 min, and the suspending portion was discarded. The cellular pellet was resuspended in the basal growth medium and centrifuged at 1200 rpm for 5 min again. Finally, the resuspension solution was filtered through a 40 *μ*m cell strainer (BD Falcon) to remove debris, and the remaining cells were plated onto a 6-well tissue culture plate (Corning) and were incubated in a humidified atmosphere at 37°C with 5% CO_2_. After 48 h, the medium was replaced to remove the nonadherent cells and then was changed every 3 days thereafter. Once the adherent cells reached 80–90% confluence, they were split with 0.25% trypsin/0.02% ethylenediamine tetraacetic acid (EDTA; Genom) and were passaged at a dilution of  1 : 3. All the cells used in the experiments were obtained from passages 3 to 10.

### 2.2. Flow Cytometry Analysis of Human ADSCs

To clarify the isolated human ADSCs, the third-passage cells were used for flow cytometry analysis. Briefly, the adherent cells were harvested by trypsinization when reaching 80–90% confluence, centrifuged at 800 rpm for 6 min, washed with sterile PBS, and resuspended at 10^6^ cells/mL. Cells were stained with specific fluorescein isothiocyanate-conjugated monoclonal antibodies against human CD14, CD29, CD31, CD45, and CD90 for 45 min at 4°C. All primary antibodies were obtained from eBioscience. For the negative control, same-isotype irrelevant antibody was used. Finally, the cells were analyzed using a FACS Calibur cytometer (BD).

### 2.3. Osteogenic Differentiation

Human ADSC osteogenic differentiation medium was purchased from Cyagen, and culturing procedures were performed in accordance with the manufacturer instructions. Briefly, when human ADSCs reached approximately 80–90% confluence, they were carefully aspirated off the basal growth medium, transferred to osteogenic differentiation medium, and cells were refed every 3 days. After 2-3 weeks of differentiation, cells were stained with Alizarin Red: cells were removed from the osteogenic differentiation medium, fixed with 4% formaldehyde solution for 30 min, and stained with Alizarin Red working solution for 3–5 min. Then, the cells were visualized under a light microscope (Olympus), and images were captured for more detailed analysis.

### 2.4. Adipogenic Differentiation

Human ADSC adipogenic differentiation medium was also purchased from Cyagen, which includes two media, medium A and medium B. When human ADSCs reached 100% confluence or were postconfluent, the old basal growth medium was discarded and replaced with medium A. Three days later, the medium was replaced with medium B. Twenty-four hours later, the medium was changed back to medium A. This cycle was repeated for optimal differentiation of human ADSCs into adipogenic cells. When large numbers of small lipid droplets appeared in the cells, the cells were cultured in medium B for an additional 3–5 days replacing the medium every 3 days. After differentiating, cells were stained with Oil Red O. Briefly, the adipogenic differentiation medium was removed, and the cells were fixed with 4% formaldehyde solution for 30 min and stained with Oil Red O working solution (3 : 2 dilution with distilled water and filtered with filter paper) for 30 min. Finally, the cells were visualized under a light microscope (Olympus), and images were captured.

### 2.5. Generation of Neurospheres from Human ADSC

Human ADSCs were induced into neural lineages as described previously [16]. Briefly, when human ADSCs were approximately 80–90% confluent, they were harvested and plated in 60 mm tissue culture dishes without any attachment factor at a concentration of 5 × 10^5^ cells/mL in neurobasal medium (Gibco) with 20 ng/mL human epithelial growth factor (hEGF; Peprotech), 20 ng/mL basic fibroblast growth factor (bFGF; Peprotech), and 2% B27 (50×; Gibco) in a 37°C humidified 5% CO_2_ atmosphere to achieve the formation of floating bodies. The floating bodies, considered as the “neurospheres,” are cell aggregates arranged in three-dimensional structures. Cells were added to the aforementioned factors every 2 days for up to 4–6 days.

### 2.6. Terminal Differentiation of Neurospheres

For terminal differentiation, these so-called neurospheres were then seeded on a 6-well poly-d-lysine tissue culture plate (Corning) in neurobasal medium (Gibco) supplemented with 2% B27 (50×; Gibco), 1% FBS (Gibco), and 1x P/S (Penicillin/Streptomycin). The medium was changed every 3-4 days and cells were incubated for 2-3 weeks. Under these conditions, the terminal differentiated cells were harvested.

### 2.7. Immunocytochemistry

Cells, including human ADSCs, neurospheres, and the terminal differentiated cells of neurospheres, were subjected to immunocytochemistry. Cells were seeded on poly-l-lysine-coated cover slips, fixed with 4% paraformaldehyde for 30 min, permeabilized using 0.25% Triton X-100 (Amresco, diluted in PBS) for 18 min, incubated with 5% bovine serum albumin (Solarbio, diluted in PBS) for 60 min at room temperature, and reacted overnight at 4°C with the following antibodies: rabbit-anti-Nestin (1 : 150 diluted in PBS; Sigma), rabbit-anti-GFAP (1 : 1000 diluted in PBS; Abcam), rabbit-anti-MAP-2 (1 : 50 diluted in PBS; Abcam), and mouse-anti-NeuN (1 : 50 diluted in PBS; Milipore). Then, cells were incubated for 60 min with secondary antibody, either Cy3-conjugated goat anti-rabbit (1 : 100 diluted in PBS; Peprotech) or TRITC-conjugated goat anti-mouse (1 : 100 diluted in PBS; Peprotech), and were labeled with 4′,6-Diamidino-2-phenylindole dihydrochloride (1 : 100 diluted in PBS; Sigma) for 5 min. Immunostaining patterns were visualized with a fluoromicroscope (Olympus).

For cell proliferation studies, human ADSCs and neurospheres were stained with rabbit anti-Ki-67 (1 : 1000 diluted in PBS; Abcam) according to the procedure described above.

### 2.8. qRT-PCR

Total RNA was extracted from undifferentiated or differentiated human ADSCs. Then the same amount of total RNA was transcribed into cDNA using oligo(dT), primers, and reverse transcriptase (M-MLV; Takara, Japan). Real-time PCR was performed using a PCR Thermocycle Instrument (Illumina eco) with SYBR Green/Fluorescein qPCR Master Mix (2x) (Fermentas), each primer, and cDNA for each reaction. The primers used for human Nestin, GFAP, NeuN, and MAP2 were designed by Jinsirui Company (China). Specific primer sequences (forward and reverse) are listed in Table 1. The amplification conditions were as follows: 50°C for 2 min and 95°C for 10 min, followed by 40 cycles of 95°C for 30 s and 60°C for 30 s. The amount of the target sequence was normalized to that of the housekeeping gene *β*-actin. For analyzing relative gene expression, the comparative Ct method (2^−ΔΔCt^) was used.

### 2.9. Western Blotting

Before and after the differentiation of human ADSCs into neurospheres, total cells were washed in sterile PBS and were lysed in RIPA Lysis Buffer (Beyotime; China). The same amount of protein in each lysate was separated by denaturing polyacrylamide gel electrophoresis and was analyzed using standard immunoblotting protocols for the expression of human Nestin, GFAP, NeuN, and MAP2. Expression of *β*-actin was used as a qualitative control to normalize the protein levels for the Western blot procedure. Human ADSCs were also analyzed as a negative control.

### 2.10. Cell Cycle Analyses

After 72 h culture, neurospheres were harvested and fixed in iced 75% ethanol overnight. Then, cells were resuspended in 250 *μ*L PBS containing 2 ug RNaseA solution (diluted in water) and were treated for 40 min at 37°C in a humidified 5% CO_2_ atmosphere. Five ug propidium iodide (PI, diluted in PBS) was added to the solution and incubated for 20 min at room temperature in the dark. The relative DNA content and the distribution of the cell population at the G1-, S-, G2-, and M-phase of the cell cycle were quantified by flow cytometry. In addition, the tests were performed after dissociating neurospheres into single cells.

### 2.11. Statistical Analysis

The experiments were repeated independently at least three times. One-way ANOVA analyses were used. Data are expressed as means ± SDs. A value of *P* < 0.05 was considered statistically significant.

## 3. Results

### 3.1. Isolation and Characterization of Human ADSCs

**Figure 1:**
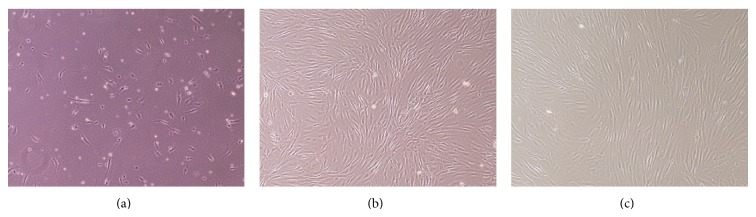
Human ADSCs expanded to a spindle-shaped fibroblast-like morphology. Phase contrast images at 40x magnification are shown. (a) Cells adhered to the plastic surfaces within 48 h. (b) Cells approached confluence when cultured for 9-10 days. (c) Cells cultured for up to 15 passages without major morphological alterations.

The human ADSCs were isolated from the abdominal subcutaneous adipose tissue of 5 donors by collagenase digestion. When human ADSCs were planted on tissue culture plates, they adhered to the plastic surfaces and exhibited a spindle-shaped morphology (Figure 1(a)). As the cells approached confluence within 9-10 days of culture, they showed a homogenously flat, fibroblast-like morphology (Figure 1(b)). The cells propagated rapidly in vitro and were passaged every 3-4 days to a maximum of 15 passages without major morphological alterations (Figure 1(c)).

**Figure 2:**
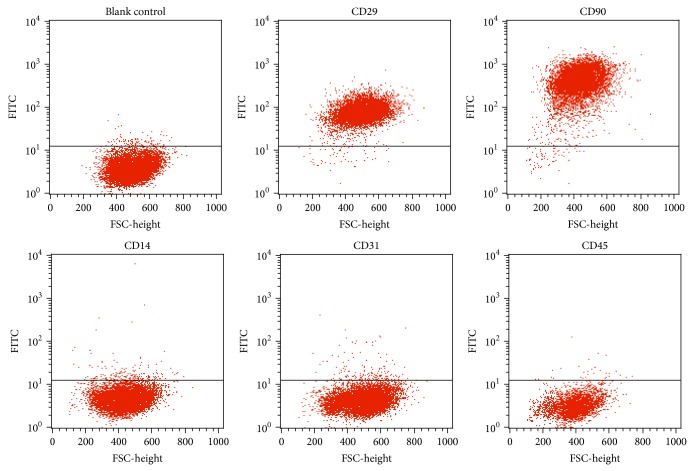
Cell surface markers of human ADSCs. Human ADSCs were labeled with FITC-conjugated antibodies specific to CD14, CD29, CD31, CD45, and CD90. Flow cytometry analysis showing that human ADSCs expressed the mesenchymal stem cell markers CD29 (92.42%) and CD90 (82.62%) but did not express the hematopoietic markers CD14, CD31, and CD45.

To further characterize human ADSCs, cell surface markers of passage-three cells were examined by flow cytometry. Flow cytometry analysis revealed that the cells showed high expression of the characteristic MSC markers CD29 and CD90 (92.42% and 82.62%, resp.) and low expression of the hematopoietic markers CD14, CD31, and CD45 (1.94%, 1.79%, and 0.33%, resp.) (Figure 2).

### 3.2. Adipogenic and Osteogenic Differentiation of Human ADSCs

To determine whether human ADSCs are multipotent, cells were cultured in selection media. Since human ADSCs did not spontaneously differentiate into various cell types in vitro during culture expansion, differentiation into adipocytes and osteoblasts was carried out in specific media and was confirmed by specific staining.

**Figure 3:**
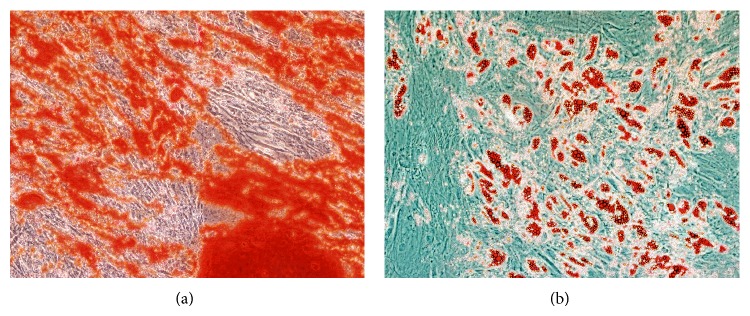
Adipogenic and osteogenic differentiation of human ADSCs in vitro. (a) Osteogenic differentiation of human ADSCs was confirmed by Alizarin Red staining, indicating positive reaction of calcium nodules. (b) Adipogenic differentiation of human ADSCs was confirmed by Oil Red O staining, showing a large amount of lipid deposition. Phase contrast images at 100x magnification are shown.

When human ADSCs were cultured in osteogenic medium for 6 days, cells reached 100% confluence and some granules were observed in or outside the cytoplasm. Within 9 days, cells showed colony growth, the granule amount increased, and calcium deposits were observed between cells. At 13 days, large calcium nodules were formed, and cells located at the center of the calcium nodules gradually lost their structure; at the same time, more floating secretions were visible in the medium. At 19 days, Alizarin Red staining showed positive reaction of the calcium nodules (Figure 3(a)).

After adipogenic induction for 72 h, small, bubble-shaped lipid droplets appeared in a portion of the cells. One week later, the cell morphology changed from a long, fibroblast-like shape into a round or polygonal shape, with the amount and size of lipid droplets increasing. After 9 days, the cells fixed by Oil Red O staining showed a large amount of lipid deposition (Figure 3(b)).

### 3.3. Generation and Differentiation of Neurospheres from Human ADSCs

**Figure 4:**
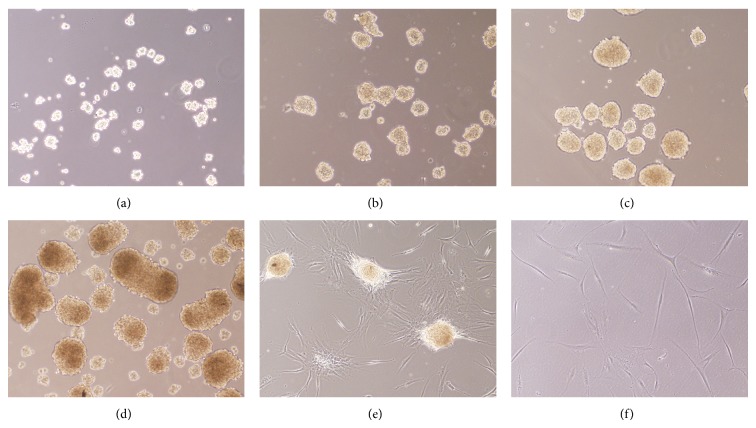
Generation and differentiation of neurospheres from human ADSCs. (a) Human ADSCs were planted in a low-attachment culture dish in neurosphere culture medium at high density, and neurospheres gradually became organized within a few hours. (b) The number of neurospheres increased over 24 h. (c) After 72 h, the large sphere clumps became dark in the center. (d) After 5 days, the spheres became even darker. (e) Neurospheres were seeded onto poly-d-lysine culture dishes and were cultured in the neurogenic medium and, 2 days later, the cell mass adhered to and spread across the growth surface. (f) At 14 days, most of the cells in neurospheres showed progressively extended long processes similar to typical neural shapes. Phase contrast images at 100x magnification are shown.

When human ADSCs were planted in a low-attachment culture dish in neurosphere culture medium at high density, the neurospheres growing in the suspension became organized within a few hours (Figure 4(a)). Over days of culture, the size of neurospheres increased (Figure 4(b)). When the spheres formed, they were frequently observed clumped together. Within 3-4 days, the large clumps of spheres became dark in the center, indicating that several cells were dying and that the neurospheres were ready to be passaged (Figure 4(c)). If left in culture for 2-3 more days, an increased number of cells died and the spheres became darker (Figure 4(d)). The floating neurospheres were passaged by harvesting and centrifuging the medium. Then, the neurospheres were resuspended in fresh neurosphere culture medium and dissociated by pipetting in trypsin/EDTA to obtain single cells, which were seeded in a new culture dish.

For terminal differentiation, neurospheres were seeded onto poly-d-lysine culture dishes and cultured with the neurogenic medium. Within 2 days of culture, the cell mass began to adhere to and spread across the growth surface (Figure 4(e)). After 14 days, the majority of cells progressively underwent morphological changes similar to typical neural shapes, forming a contracted multipolar cell body with long processes (Figure 4(f)).

### 3.4. Characterization of Cells Differentiated from Neurospheres

**Figure 5:**
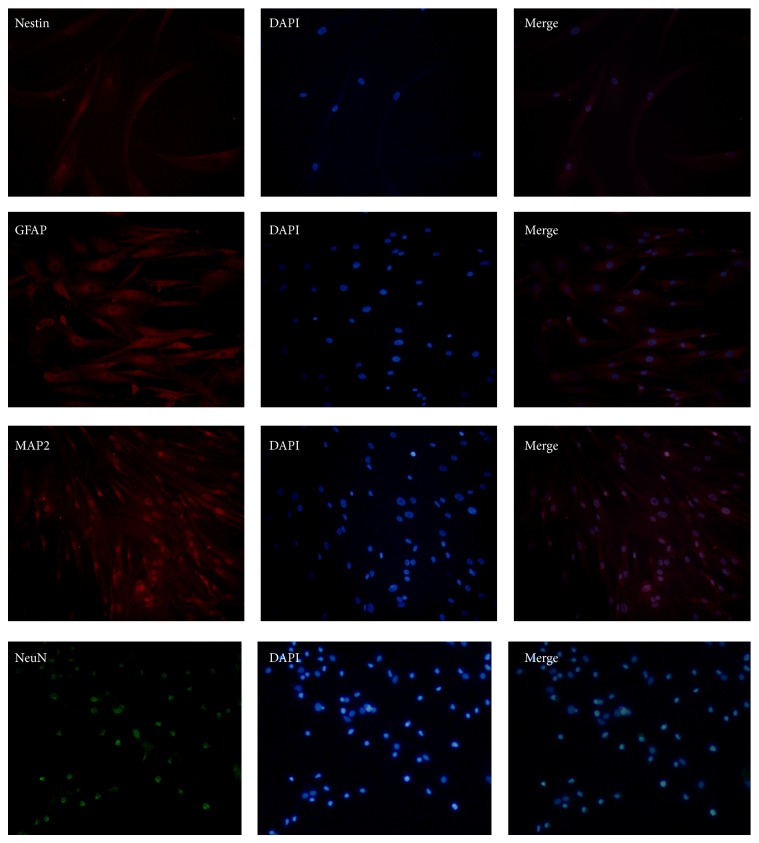
Immunocytochemistry for the expression of Nestin, GFAP, MAP2, and NeuN in cells differentiated from neurospheres. Analyses showed that the cells were positive for the expression of these markers, especially the expression of the mature neural marker (NeuN) and astrocyte marker (GFAP). Phase contrast images at 200x magnification are shown.

**Figure 6:**
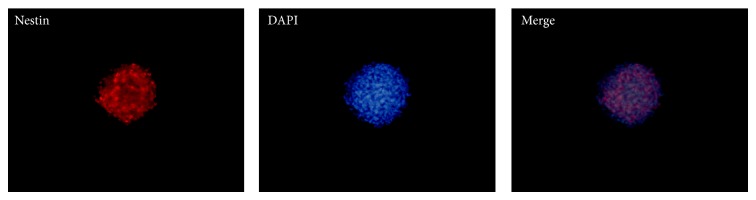
Immunocytochemistry showed high levels of Nestin by the neurospheres. Phase contrast images at 100x magnification are shown.

**Figure 7:**
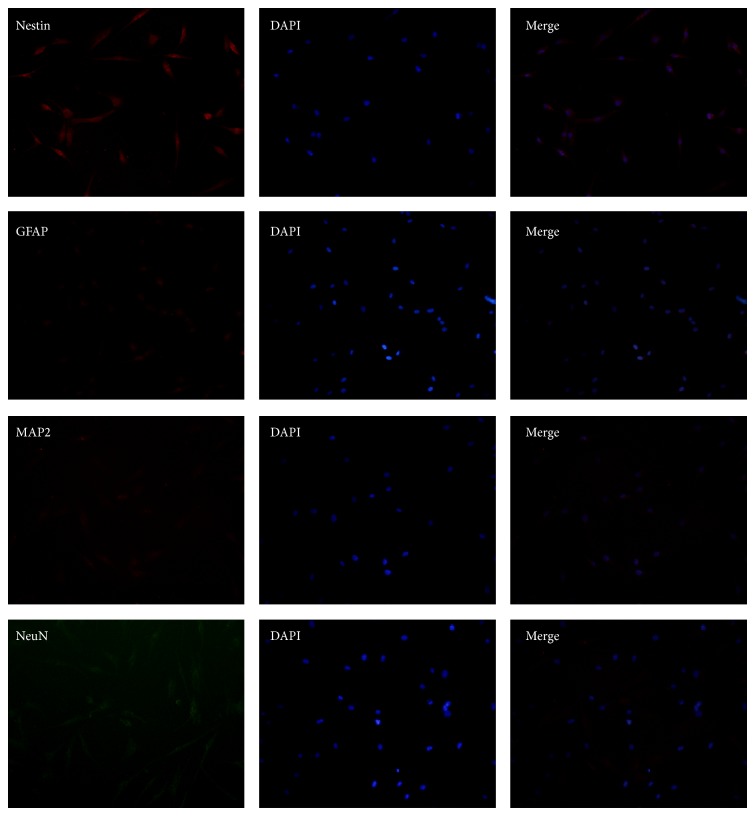
Immunocytochemistry for the expression of Nestin, GFAP, MAP2, and NeuN in human ADSCs. Results showed human ADSCs only expressed low levels of Nestin, and the expression of other markers was undetectable. Phase contrast images at 200x magnification are shown.

To further characterize neural-linage differentiated capability of neurospheres, cells were stained with NeuN, MAP2, GFAP, and Nestin. The cells were positive for (NeuN) and astrocyte marker (GFAP) (Figure 5). However, the expressed levels of Nestin were reduced compared with neurospheres, which expressed high levels of Nestin (Figure 6). In contrast, human ADSCs only expressed low levels of Nestin, and the expression of other markers was undetectable (Figure 7).

**Figure 8:**
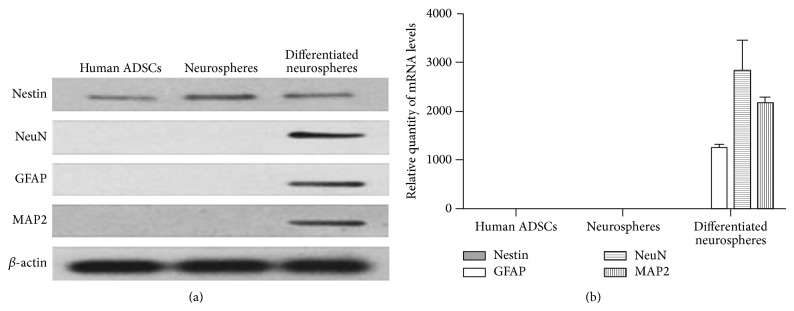
The expression of Nestin, MAP2, NeuN, and GFAP in cells. (a) Western blotting analysis demonstrated that MAP2, NeuN, and GFAP protein levels in the cells differentiated from neurospheres were upregulated, whereas Nestin expression levels were downregulated compared to those of neurospheres, and the expression of Nestin was the highest in neurospheres. (b) The results from qRT-PCR matched the Western blotting findings: the neural cells showed high expression of MAP2, NeuN, and GFAP at the mRNA level.

Western blotting analysis demonstrated that MAP2, NeuN, and GFAP protein levels in the cells differentiated from neurospheres were upregulated, whereas Nestin expression levels were downregulated compared to those of neurospheres (Figure 8(a)). As a negative control, protein levels of human ADSCs and neurospheres were also examined, and both cell types expressed Nestin but not NeuN, MAP-2, or GFAP (data not shown). The expression of Nestin was higher in neurospheres than in the other two cell types (Figure 8(a)).

qRT-PCR analysis confirmed that only the cells differentiated from neurospheres were positive expression of MAP2, NeuN, and GFAP mRNA. By contrast, both the cells, including human ADSCs and neurospheres, showed positive expression of Nestin mRNA (Figure 8(b); levels are relatively too low for visual comparison). Human ADSCs and neurospheres were absent of NeuN, GFAP, and MAP2 mRNA (Figure 8(b)). Therefore, the qRT-PCR results were in line with the Western blotting findings.

### 3.5. Cell Proliferation in Neurospheres

**Figure 9:**
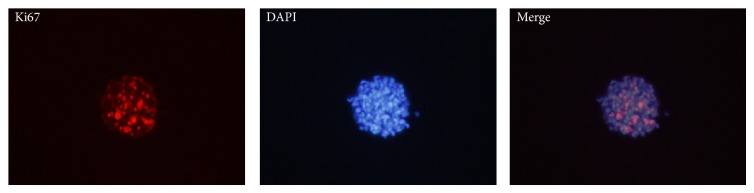
Cell proliferation in neurospheres. Immunofluorescence revealed positive Ki67 expression in some neurospheres. Phase contrast images at 200x magnification are shown.

Immunofluorescence revealed clear positive expression of Ki67, a cell proliferation marker, in some cells of neurospheres from human ADSCs (Figure 9).

**Figure 10:**
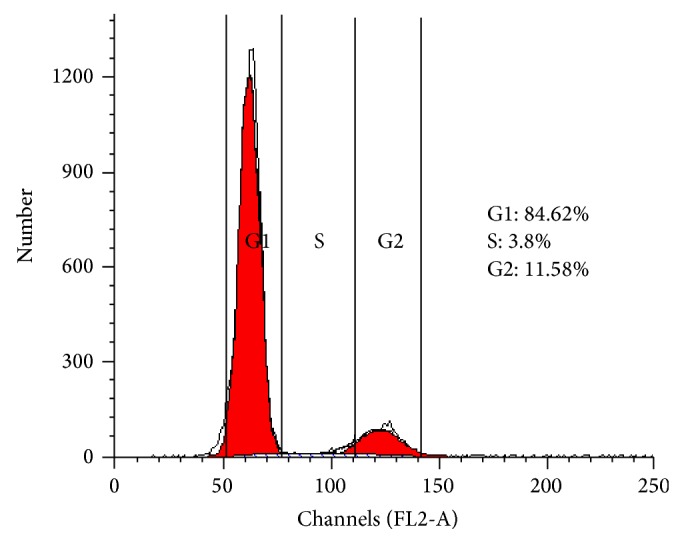
Cell cycle analysis of neurospheres. Flow cytometry analysis of the cell cycle showed that 15.38% of cells in neurospheres were in the G2/M phase, 14.08 ± 8.74% were in the S phase, and the remaining cells were in the G0/G1 phase (84.62%).

Cell cycle refers to the cell from a split finish to the next split end going through the same activities repeatedly (i.e., G1 phase → S phase → G2 phase → M phase). A cell cycle contains mitosis phase (M phase) and the interphase mitosis (including G1 phase, S phase, and G2 phase). The cell cycle results showed that 15.38% of the cells in neurospheres were at the S + G2/M phase (active proliferative phase) with the remaining cells in the G0/G1 phase (quiescent phase, 84.62%) (Figure 10). Therefore, some cells in neurospheres were at the growth phase of the cell cycle.

## 4. Discussion

Currently, few effective treatments for many neurological disorders were discovered. The use of fetally derived neuronal stem cells is associated with ethical problems, as well as clinical issues, such as the requirement for immune suppression. ADSCs are more easily available, with neither immunoreactive nor ethical considerations.

Human ADSCs were induced to neural lineages by seeding at high density in serum-free medium enriched with a combination of EGF and bFGF. EGF and bFGF, mitogen specific growth factors, were regarded as key factors to isolate NSCs from neural tissue based on the original work in this field [17], in which cell division and proliferation were not observed in the absence of EGF. In addition, the production of neurospheres from human ADSCs requires a culture substrate without any attachment factor [18]. A large amount of neurospheres derived from human ADSCs was generated in vitro, whose cellular morphology was similar to NSCs isolated from the brain and spinal cord, grown in culture as free-floating aggregates [4]. ADSCs-derived neurospheres, like NSCs derived from embryonic and adult brain tissues, expressed high levels of Nestin [19]. Nestin, a marker of neural stem and progenitor cells, is expressed during the early developmental stages in the central nervous system and peripheral nervous systems. Upon differentiation, Nestin becomes downregulated and is replaced by other tissue-specific proteins [20]. The expression pattern of Nestin in our study was confirmed by immunocytochemistry, qRT-PCR, and Western blotting. High levels of Nestin in ADSCs-derived neurospheres were downregulated in terminal differentiated cells form neurospheres.

In the culture systems, removal of mitogens and the addition of serum would result in the differentiation of neurospheres derived from ADSCs into neurons- and glia-like cells [13]. Ahmadi et al. discovered that cells adhered showing long chains of cellular processes and were positive for Nestin, MAP2, and GFAP, when ADSCs-derived neurospheres were seeded onto an adherent surface after growth factors withdrawal [21]. Razavi et al. found ADSCs-derived neurospheres could further differentiat into Schwann-like cells based on their production of neurospecific proteins including S100 and GFAP [22]. In the present study, we detected the cells differentiated from ADSCs-derived neurospheres for the expression of neurospecific markers (Nestin, MAP2, NeuN, and GFAP) by immunostaining, Western blotting, and qRT-PCR; the results showed these cells were positive for the expression of the markers, especially the expression of MAP2, NeuN, and GFAP. MAP2 and NeuN are markers of mature neurons, whereas GFAP is a protein specifically found in mature glial cells.

In the next study, the functional characteristics of neurons and glia cells including electrophysiological recordings and ion channel expression should be examined. Furthermore, the potential clinical utility of ADSCs-derived neurospheres should be evaluated by performing transplantation experiments.

Additional studies are performed by whether ADSCs-derived neurospheres could proliferate in vitro. Immunofluorescence revealed that some of the cells in neurospheres showed clear positive expression of Ki67, a nuclear antigen associated with cell proliferation. The cell cycle analyses of neurospheres showed 15.38% of cells in neurospheres in the active proliferative phase. These results show that ADSCs-derived neurospheres can proliferate in vitro.

Recent studies have reported that hormones including estrogen can stimulate proliferation in mouse ESCs and affect the differentiation capacities of human MSCs; reports have also shown the presence of estrogen and testosterone receptors on stem cells, suggesting that estrogen and testosterone may modify the function of those cells [23, 24]. In an example of sex steroid-specific effects, neural stem cells exhibit enhanced proliferation in response to estrogens while androgens mediate inhibitory effects on their proliferation [25].

In our study, we concluded that plentiful generation of neurospherers from ADSCs mainly owed to the effective inducing technique; however, we could not rule out whether the gravidae-derived ADSCs proliferate much faster because of the hormones. Therefore, advanced research should be performed to compare the proliferation capacity of ADSCs and ADSCs-derived neurospheres among various population sources especially between the gravidae and nulligravida.

To summarize, the results of this study demonstrated that human ADSCs have the capacity to efficiently generate neurospheres which expressed high levels of the neural stem cell marker Nestin on uncoated culture disks. And the differentiated cells of neurospheres were positive for the expression of Nestin, MAP2, NeuN, and GFAP, especially the expression of MAP2, NeuN, and GFAP. In addition, some cells in ADSC-derived neurospheres were in the active proliferative phase. The properties of ADSC-derived neurospheres were similar to NSCs derived from embryonic and adult brain tissues.

Thus, neurospheres derived from ADSCs offer a more clinically feasible source than brain-derived neural stem cells. And human ADSCs-derived neurospheres may be an ideal alternative source of stem cells for the treatment of neurodegenerative diseases.

## Figures and Tables

**Table 1:** Primer sequences used for RT-PCR.

Gene	Forward (top) and reverse (bottom)	Size (bp)
Nestin	5′-ATAGAGGGCAAAGTGGTAAGCAG-′3	177
	5′-TTCTAGTGTCTCATGGCTCTGGTT-′3	

GFAP	5′-GGAAGATTGAGTCGCTGGAG-3′	164
	5′-ATACTGCGTGCGGATCTCTT-3′	

MAP2	5′-CATACAGGGAGGATGAAGAGGG-3′	268
	5′-GGTGGAGAAGGAGGCAGATTAG-3′	

NeuN	5′-GCGGCTACACGTCTCCAACATC-3′	189
	5′-ATCGTCCCATTCAGCTTCTCCC-3′	

*β*-actin	5′-CACGATGGAGGGGCCGGACTCATC-3′	240
	5′-TAAAGACCTCTATGCCAACACAGT-3′	
